# Is D2 Lymphadenectomy Alone Suitable for Gastric Cancer With Bulky N2 and/or Para-Aortic Lymph Node Metastases After Preoperative Chemotherapy?

**DOI:** 10.3389/fonc.2021.709617

**Published:** 2021-09-02

**Authors:** Wei Xu, Wentao Liu, Lingquan Wang, Changyu He, Sheng Lu, Zhentian Ni, Zichen Hua, Zhenglun Zhu, Birendra Kumar Sah, Zhongyin Yang, Yanan Zheng, Runhua Feng, Chen Li, Xuexin Yao, Mingmin Chen, Chao Yan, Min Yan, Zhenggang Zhu

**Affiliations:** Department of General Surgery, Shanghai Key Laboratory of Gastric Neoplasms, Shanghai Institute of Digestive Surgery, Ruijin Hospital, Shanghai Jiao Tong University School of Medicine, Shanghai, China

**Keywords:** gastric cancer, preoperative chemotherapy, bulky N2 metastases, para-aortic lymph node metastases, D2 lymphadenectomy

## Abstract

**Background:**

For gastric cancer (GC) with extensive lymph node metastasis (bulky N2 and/or para-aortic lymph node metastases), there is no standard therapy worldwide. In Japan, preoperative chemotherapy (PCT) followed by D2 gastrectomy plus para-aortic lymph node dissection (PAND) is considered the standard treatment for these patients. However, in China, the standard operation for GC patients with only bulky N2 metastases was D2 gastrectomy. Besides, after PCT, whether doing PAND improves survival or not is debatable for GC patients with para-aortic lymph node (PAN) metastases. Therefore, we conducted this study to investigate whether D2 lymphadenectomy alone is suitable for these patients after PCT.

**Methods:**

We retrospectively collected data on patients from our electronic medical record system. GC patients with bulky N2 and/or PAN metastases who underwent D2 lymphadenectomy alone after PCT were enrolled. The survival outcomes and chemotherapy responses were analyzed and compared with the results of the JCOG0405 study.

**Results:**

From May 2009 to December 2017, a total of 83 patients met all eligibility criteria and were enrolled. The median survival duration for all patients was 40.0 months. The 3-year and 5-year OS rates for all patients were 50.3% and 45.6%, respectively. For patients with only bulky N2 metastasis, the 3-year and 5-year OS rates were 77.1% and 71.6%, respectively, which were similar to the results of the JCOG0405 study (82.7% and 73.4%). For patients with only PAN metastases, the 3-year and 5-year OS rates were 50.0% and 50.0%, respectively, which seemed to be lower than those of the JCOG0405 study (64.3% and 57.1%). For patients with bulky N2 and PAN metastases, the 3-year and 5-year OS rates were 7.4% and 0.0%, respectively, which were lower than those of the JCOG0405 study (20.0% and 20.0%).

**Conclusion:**

The results of our study suggest that D2 lymphadenectomy alone is suitable for GC patients with only bulky N2 metastasis after PCT. However, D2 lymphadenectomy alone perhaps is not suitable for patients with bulky N2 and PAN metastases after PCT.

## Introduction

Gastric cancer (GC) is the fifth most common cancer and the third leading cause of cancer-related deaths worldwide (1). Surgery is the most effective and basic treatment for GC (2). Radical surgery includes gastrectomy and lymphadenectomy. Based on the 15-year results of a Dutch trial, D2 lymphadenectomy is considered the standard treatment for GC (3). Currently, most guidelines, including those established by the ESMO (4), NCCN (5), JGCA (6) and CSCO (7), have introduced D2 lymphadenectomy as the standard surgical procedure for GC. For advanced gastric cancer (AGC), however, the efficacy of surgery alone is limited. In recent years, more attention has been given to comprehensive treatment, particularly perioperative chemotherapy (preoperative and postoperative chemotherapy) (8–11). There is a special group of patients who have bulky N2 and/or para-aortic lymph node (PAN) metastases. Eastern and Western scholars have different opinions on the treatment of these patients. A bulky nodal lesion surrounding the coeliac artery and its branches with a diameter ≥ 3 cm or at least two adjacent tumors ≥ 1.5 cm are defined as bulky N2 metastases by the Japanese scholars (12, 13). In addition, PAN with a diameter ≥ 1 cm is considered PAN metastases (12, 13). The tumor node metastases (TNM) staging system considers PAN as distant metastases (M1) (14).

**Figure 1:**
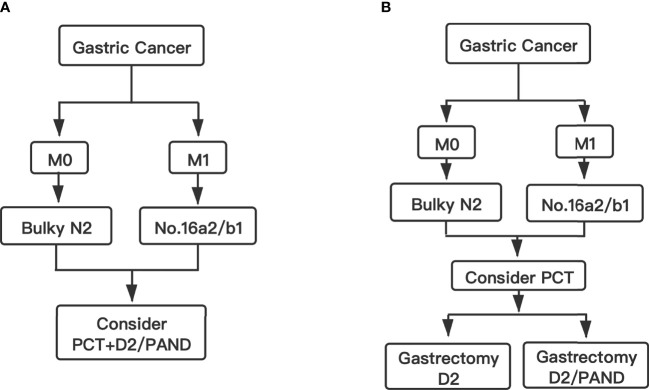
Flow chart. **(A)** The standard therapy for GC patients with bulky N2 and/or PAN metastases in Japan. **(B)** The conclusion and suggested therapy for GC patients with bulky N2 and/or PAN metastases in this study.

In Western countries, bulky N2 and/or PAN metastases are considered unresectable and warrant palliative chemotherapy. These patients can hardly survive for more than 3 years with chemotherapy alone or noncurative surgery followed by chemotherapy (15). Some previous studies showed that the addition of gastrectomy to chemotherapy might improve patient survival (median overall survival of 8.0-12.2 months with gastrectomy vs 2.4-6.7 months without gastrectomy) among GC patients with a single non-curable factor (16–23). The prognosis of these patients is still poor. In order to improve the prognosis of these patients, Japanese scholars conducted several studies to investigate new treatment strategies (12, 13, 24). The JCOG0001 study was the first clinical trial to evaluate the efficacy and safety of preoperative chemotherapy (PCT) followed by gastrectomy with D2 lymphadenectomy plus PAN dissection (PAND) for GC patients with bulky N2 and/or PAN metastasis (13). Although the JCOG0001 study was terminated because of a high number of treatment-related deaths, it provided a promising 3-year survival rate (27%). With the improvement of the PCT regimen, a similar study was conducted (JCOG0405) (12). The 3-year and 5-year survival rates in the JCOG0405 study were 59% and 53%, respectively. The study showed that PCT with the CS regimen (cisplatin and S-1) followed by gastrectomy with D2 lymphadenectomy plus PAND was safe and effective for GC patients with extensive lymph node metastasis. Recently, the JCOG1002 study also investigated the same subject with a different PCT regimen (DCS: docetaxel, cisplatin, and S-1) (24). The 3-year and 5-year survival rates in the JCOG1002 study were 62.7% and 54.9%, respectively, which were similar to those in the JCOG0405 study. At present, the standard therapy for these patients in Japan is still that stated in the JCOG0405 protocol. All three studies (JCOG0001, JCOG0405 and JCOG1002) combined PCT with D2 gastrectomy plus PAND. Therefore, the Japanese gastric cancer treatment guidelines suggested PCT with D2 gastrectomy plus PAND is the standard therapy for GC patients with bulky N2 and/or PAN metastases (**Figure 1A**) (6).

However, in China, for GC patients with only bulky N2 metastases, the standard treatment is D2 gastrectomy without PAND (7). Besides, for GC patients with PAN metastases, a phase II trial conducted by Chinese scholars showed that PCT with XELOX (capecitabine and oxaliplatin) followed by D2 gastrectomy alone also had a sufficient R0 resection rate (25). In addition, a real-world study conducted in China concluded that, for GC patients with PAN metastases that responds well to PCT, D2 gastrectomy alone is safe and effective (26).

For GC patients with bulky N2 and/or PAN metastases, it is unclear whether, on the basis of PCT, the addition of PAND would further improve prognosis compared to D2 lymphadenectomy alone. Therefore, we conducted this study to investigate whether D2 lymphadenectomy alone is suitable for GC patients with bulky N2 and/or para-aortic lymph node metastases after PCT.

## Materials And Methods

### Patient Selection

Data on patients who were admitted to Ruijin Hospital (Shanghai Jiao Tong University School of Medicine, Shanghai, China) between May 2009 and December 2017 were collected retrospectively from the electronic medical record system. Eligibility criteria were as follows: (1) histologically proven gastric adenocarcinoma; (2) bulky N2 metastases and/or PAN metastases (Stations No. 16a2/16b1) confirmed by multidetector computed tomography (MDCT); (3) no distant metastases except for PAN confirmed by MDCT; (4) no history of other cancers; (5) patients received PCT before surgery; and (6) patients underwent D2 gastrectomy without PAND. According to the status of extensive lymph node metastases, all enrolled patients were divided into three groups: the Bulky N2+/PAN- group (only bulky N2 metastases without PAN metastases), the Bulky N2-/PAN+ group (only PAN metastases without bulky N2 metastases) and the Bulky N2+/PAN+ group (both bulky N2 and PAN metastases) group. This study was performed with approval from the Ethics Committee of Ruijin Hospital affiliated to the Shanghai Jiao Tong University School of Medicine. All patients were enrolled after signing an informed consent form.

### Preoperative Chemotherapy

The preoperative chemotherapy regimen for all patients was EOX (epirubicin, oxaliplatin and capecitabine) (27). Epirubicin 50mg/m^2^ and oxaliplatin 130mg/m^2^ were administered on day 1, repeated every 3 weeks. Capecitabine (625mg/m^2^) was given orally twice daily for the first two weeks of a 3-week cycle. Most patients received an average of three cycles of EOX chemotherapy before the imaging evaluation. A few patients received additional cycles. The National Cancer Institute Common Terminology Criteria for Adverse Events (CTCAE, 4.0) was applied for the evaluation of adverse effects.

### Imaging Evaluation

The Response Evaluation Criteria In Solid Tumors (RECIST 1.1) was used to evaluate response of PCT in this study (28). The tumor responses were divided into 4 grades: complete response (CR), partial response (PR), stable disease (SD), and progressive disease (PD).

### Surgery

All the operations were performed by the same surgical team of the gastric cancer specialized group in Ruijin hospital. For all enrolled patients, we performed open surgery for D2 gastrectomy without PAND. The radical degree of the operation was classified into three grades: R0, macroscopically complete surgical resection with negative microscopic margins; R1, macroscopically complete surgical resection with positive microscopic margins; and R2; macroscopically incomplete surgical resection. Surgical complications were graded using the Clavien-Dindo Complications Classification (CDCC) (29). In this study, surgical complications of grade III or above were recorded.

### Pathological Evaluation

After the operation, the tumor specimens were evaluated pathologically. The tumor was staged in accordance with the Japanese Classifcation of Gastric Carcinoma (30). According to the proportion of tumors affected by degeneration or necrosis, the tumor regression grade (TRG) was divided into 4 degrees: grade 0, no part of the tumor affected; grade 1a, less than one-third affected; grade 1b, between one-third and two-thirds affected; grade 2, between two-thirds and the entire tumor affected; and grade 3, no residual tumor (pathological complete response, PCR) (6). The pathological evaluation was performed in the same manner as in the JCOG0001 (13), JCOG 0405 (12) and JCOG1002 (24)studies.

### Postoperative Chemotherapy and Follow-up

The postoperative chemotherapy regimens for all patients were EOX or XELOX (oxaliplatin and capecitabine). According to the patient’s postoperative physical status, we chose the three-drug regimen or the two-drug regimen. Most patients received an average of three cycles of postoperative chemotherapy. Through outpatient visits and telephone calls, we followed up all enrolled patients. Telephone calls was conducted every three months after surgery. Overall survival (OS) was measured from the date of the initial diagnosis of gastric cancer to the date of death or the last follow-up.

### Statistical Analysis

The OS curve was estimated using the Kaplan-Meier method. To analyze baseline factors between different studies, the chi-square test was used. A two-sided P value<0.05 was considered significantly different. Analyses were performed with SPSS version 26.0 (IBM Statistical Product and Service Solutions, Armonk, USA). GraphPad Prism version 8.0 (San Diego, CA, USA) was used to draw the survival curve.

## Results

### Characteristics of the Patients

Between May 2009 and December 2017, 83 patients satisfied all eligibility criteria and were enrolled in this study. The study population comprised 59 males and 24 females, with a male-to-female ratio of 2.5:1. The median age at diagnosis was 61 years (range 31-80). In this study, 44 (53.0%), 12 (14.5%) and 27 (32.5%) patients comprised in the Bulky N2+/PAN-, Bulky N2-/PAN+ and Bulky N2+/PAN+ groups, respectively. The detailed characteristics of patients in three groups were also shown in **Table 1**, including sex, age ECOG, differentiation, body mass index (BMI), tumor location, Borrmann type, clinical nodal status, the diameter of the largest lymph node (LNmax) and the type of gastrectomy. The tumor location were classified into cardia, body, antrum and whole stomach. The LNmax was measured using multi-detector-row computed tomography (MDCT). The types of gastrectomy were divided into proximal, distal, total and multiorgan resection. A total of 4 patients received multiorgan resection. One patient in the Bulky N2+/PAN- group received total gastrectomy plus distal pancreatectomy and splenectomy. Two patients in the Bulky N2+/PAN+ group received gastrectomy plus left lateral hepatic lobectomy. One patient in the Bulky N2+/PAN+ group, from whom a constrictive metastatic lesion was found in the small intestine 30cm from the proximal ileocecal colon, received gastrectomy plus partial enterectomy (approximately 5cm of small intestine was resected).

**Table 1:** Characteristics of all enrolled patients.

Characteristics	Total (N = 83)	Bulky N2+/PAN- (n = 44)	Bulky N2-/PAN+ (n = 12)	Bulky N2+/PAN+ (n = 27)
**Sex**				
Male	59 (71.1%)	32 (72.7%)	9 (75.0%)	18 (66.7%)
female	24 (28.9%)	12 (27.3%)	3 (25.0%)	9 (33.3%)
**Age (y)**				
Median (range)	61 (31-80)	63.5 (31-80)	58.5 (40-74)	61 (43-76)
**ECOG**				
0	80 (96.4%)	41 (93.2%)	12 (100.0%)	27 (100.0%)
1	3 (3.6%)	3 (6.8%)	0 (0.0%)	0 (0.0%)
**Differentiation**				
Differentiated	41 (49.4%)	22 (50.0%)	6 (50.0%)	13 (48.1%)
Undifferentiated	42 (50.6%)	22 (50.0%)	6 (50.0%)	14 (51.9%)
**BMI (kg/m^2^)**				
Median (range)	22.3 (17.0-31.7)	22.7 (17.0-30.5)	21.8 (18.1-31.7)	22.2 (17.2-27.8)
**Location (n[%])**				
Cardia	21 (25.3%)	16 (36.4%)	3 (25.0%)	2 (7.4%)
Body	20 (24.1%)	6 (13.6%)	5 (41.7%)	9 (33.3%)
Antrum	32 (38.6%)	19 (43.2%)	3 (25.0%)	10 (37.0%)
Whole stomach	10 (12.0%)	3 (6.8%)	1 (8.3%)	6 (22.2%)
**Borrmann (n[%])**				
I	3 (3.6%)	2 (4.5%)	1 (8.3%)	0 (0.0%)
II	7 (8.4%)	5 (11.4%)	2 (16.7%)	0 (0.0%)
III	67 (80.7%)	36 (81.8%)	8 (66.7%)	23 (85.2%)
IV	6 (7.2%)	1 (2.3%)	1 (8.3%)	4 (14.8%)
**Nodal status**				
cN0	0 (0.0%)	0 (0.0%)	0 (0.0%)	0 (0.0%)
cN1	8 (9.6%)	6 (13.6%)	2 (16.7%)	0 (0.0%)
cN2	26 (31.3%)	21 (47.7%)	1 (8.3%)	4 (14.8%)
cN3	49 (59.0%)	17 (38.6%)	9 (75.0%)	23 (85.2%)
**LNmax (cm)**				
Median (range)	2.1 (1.0-5.1)	2.6 (1.5-5.1)	1.4 (1.0-2.0)	1.9 (1.0-4.2)
**Type of gastrectomy**				
Proximal	1 (1.2%)	1 (2.3%)	0 (0.0%)	0 (0.0%)
Distal	27 (32.5%)	17 (38.6%)	3 (25.0%)	7 (25.9%)
Total	51 (61.4%)	26 (59.1%)	8 (66.7%)	17 (63.0%)
Multiorgan	4 (4.8%)	0 (0.0%)	1 (8.3%)	3 (11.1%)

### Evaluation of Preoperative Chemotherapy

According to the CTCAE, one patient had grade 3 hematological adverse and three patients had grade 3 or 4 vomiting. Most of the hematological adverse effects and symptomatic adverse effects were acceptable for triplet chemotherapy. According to the RECIST, most patients in this study responsed well to PCT (**Table 2**).

**Table 2:** Evaluation of preoperative chemotherapy.

Characteristics	Total (N = 83)	Bulky N2+/PAN- (n = 44)	Bulky N2-/PAN+ (n = 12)	Bulky N2+/PAN+ (n = 27)
**RECIST**				
CR	0 (0%)	0 (0%)	0 (0%)	0 (0%)
PR	63 (75.9%)	38 (86.4%)	7 (58.3%)	18 (66.7%)
SD	19 (22.9%)	5 (11.4%)	5 (41.7%)	9 (33.3%)
PD	1 (1.2%)	1 (2.3%)	0 (0%)	0 (0%)
**Degree of gastrectomy**				
R0	43 (51.8%)	43 (97.7%)	0 (0%)	0 (0%)
R1	0 (0%)	0 (0%)	0 (0%)	0 (0%)
R2	40 (48.2%)	1 (2.3%)	12 (100.0%)	27 (100.0%)
**Depth of tumor invasion**				
ypT0	12 (14.5%)	11 (25.0%)	1 (8.3%)	0 (0%)
ypT1	6 (7.2%)	4 (9.1%)	2 (16.7%)	0 (0%)
ypT2	14 (16.9%)	7 (15.9%)	3 (25.0%)	4 (14.8%)
ypT3	10 (12.0%)	4 (9.1%)	2 (16.7%)	4 (14.8%)
ypT4	41 (49.4%)	18 (40.9%)	4 (33.3%)	19 (70.4%)
**Nodal status**				
ypN0	26 (31.3%)	20 (45.5%)	3 (25.0%)	3 (11.1%)
ypN1	14 (16.9%)	8 (18.2%)	4 (33.3%)	2 (7.4%)
ypN2	14 (16.9%)	8 (18.2%)	1 (8.3%)	5 (18.5%)
ypN3	29 (34.9%)	8 (18.2%)	4 (33.3%)	17 (63.0%)
**TRG**				
Grade 0	4 (4.8%)	1 (2.3%)	1 (8.3%)	2 (7.4%)
Grade 1a	12 (14.5%)	3 (6.8%)	1 (8.3%)	8 (29.6%)
Grade 1b	10 (12.0%)	8 (18.2%)	1 (8.3%)	1 (3.7%)
Grade 2	45 (54.2%)	21 (47.7%)	8 (66.7%)	16 (59.3%)
Grade 3	12 (14.5%)	11 (25.0%)	1 (8.3%)	0 (0%)

All patients underwent D2 gastrectomy without PAND. Therefore, patients with PAN metastases did not receive R0 resection. Besides, one patient in the Bulky N2+/PAN- group did not receive R0 resection. In this patient, metastatic nodules were found on the surface of the transverse colon and the root of the mesentery. Therefore, only 43 (51.8%) patients received R0 resection. An average of 37 (SD: 17.52, 95% CI: 33-41) lymph nodes were dissected in this study. For patients in the Bulky N2+/PAN-, Bulky N2-/PAN+ and Bulky N2+/PAN+ groups, the average numbers of lymph nodes dissected were 36, 31, and 41, respectively.

The pathological evaluations were also shown in **Table 2**. Patients with pathological grade ypT0 were considered to achieve a pathological complete response in the primary tumors. Patients with grade ypN0 were considered to achieve a pathological complete response in the lymph nodes. There were 11 patients received pathological complete response both in the primary tumors and lymph nodes. One patient achieved the pathological complete response in the primary tumors, but the lymph nodes did not get the pathological complete response. At last, 12 (14.5%) patients had complete tumor regression (TRG 3), and 45 (54.2%) patients had subtotal tumor regression (TRG 2) (**Table 2**).

### Surgical Complications

Only two (2.4%) patients experienced grade III or above complications after surgery. Both patients were in the bulky N2+/PAN- group and had leakage. One patient underwent a second surgery due to anastomotic leakage. Another patient with duodenal stump leakage did not undergo a second surgery. There was no treatment-related or in-hospital death.

### Survival Analysis

In this study, the last follow-up date was 22 December 2020, and the median follow-up time was 55.8 months (range 36.6–141.5 months). By the time of the last follow-up time, all patients had been followed up for 3 years and 28 patients had been followed up for 5 years. Among the 28 patients who had been followed up for 5 years, there were 20, 1, and 7 patients in the Bulky N2+/PAN-, Bulky N2-/PAN+ and Bulky N2+/PAN+ groups, respectively.

**Figure 2:**
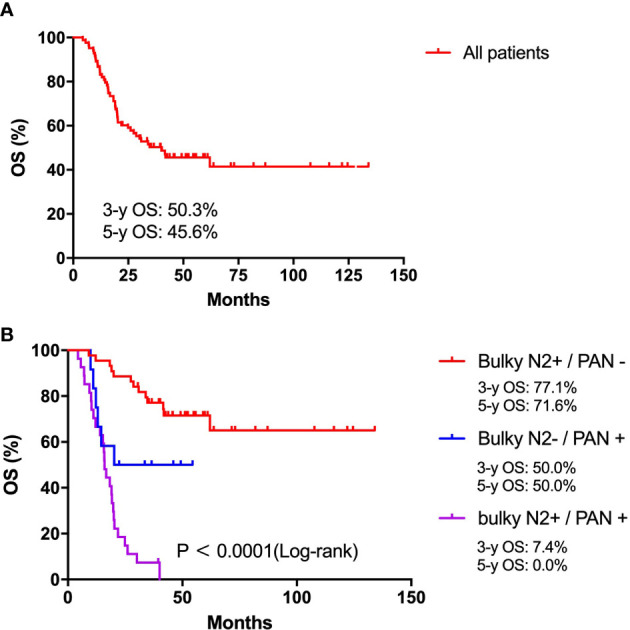
Survival analysis. **(A)** Survival analysis for all patients with bulky N2 and/or para-aortic lymph node metastases. **(B)** Survival analysis for patients between the Bulky N2+/PAN-, Bulky N2-/PAN+ and Bulky N2+/PAN+ groups.

Survival curves for patients are shown in **Figure 2**. In this study, the median survival duration for all patients was 40.0 months. The 3-year and 5-year OS rates for all patients were 50.3% and 45.6%, respectively (**Figure 2A**). The median OS duration of patients in the Bulky N2+/PAN-, Bulky N2-/PAN+ and Bulky N2+/PAN+ groups were undefined, 77.1 and 15.9 months, respectively (P<0.0001, **Figure 2B**). The 3-year and 5-year OS rates for patients in the Bulky N2+/PAN- group were 77.1% and 71.6%, respectively. The 3-year and 5-year OS rates for patients in the Bulky N2-/PAN+ group were 50.0% and 50.0%, respectively. The 3-year and 5-year OS rates for patients in the Bulky N2+/PAN+ group were 7.4% and 0.0%, respectively.

After all patients were followed up for 3 years, 40 (48.2%) patients were still alive. For patients in the Bulky N2+/PAN- group, Bulky N2-/PAN+ and Bulky N2+/PAN+ groups, there were 32 (72.7%), 5 (41.7%) and 3 (11.1%) patients still alive, respectively.

In addition, in the Bulky N2+/PAN- group, 20 patients were followed up for 5 years and 7 patients died of cancer recurrence. In the Bulky N2-/PAN+ and Bulky N2+/PAN+ groups, there were 1 and 7 patients followed up for 5 years and no one survived.

## Discussion

Currently, there is no standard treatment for gastric cancer with extensive lymph node metastases (bulky N2 and/or para-aortic lymph node metastases). In the West, these tumors are considered unresectable and tend to be treated with palliative chemotherapy. In Japan, PCT with the CS regimen followed by D2 gastrectomy plus PAND is considered the standard treatment for these tumors. Advances in research over recent years have focused only on regimen changes in PCT (12, 13, 24). However, it is unclear whether, on the basis of PCT, the addition of PAND could further improve prognosis compared to D2 lymphadenectomy alone. In China, PAND has not been widely carried out due to its highly technical difficulties, surgical complications and uncertain survival benefits. Therefore, we aimed to explore whether D2 lymphadenectomy alone is suitable for GC patients with bulky N2 and/or PAN metastases after PCT by comparing the results of our study and those from Japanese scholars.

The detailed OS rates of patients in the JCOG0001, JCOG0405, JCOG1002 studies and this study are showed in **Table 3**. The survival data were derived from analysis of 49 patients in JCOG0001 and 47 patients in JCOG0405 who underwent surgery, 52 eligible patients in JCOG1002 and all patients in this study. Integrated analysis demonstrated that the results of the JCOG0405 study were better than those of the JCOG0001 study for GC with extensive lymph node metastasis (31). In addition, the long-term outcomes of the JCOG1002 study also demonstrated that PCT with the CS regimen followed by D2 gastrectomy plus PAND remains the standard treatment for patients with extensive nodal metastases in Japan (32). Therefore, we mainly compared our study with the JCOG0405 study, in which 49 (92.5%) patients underwent surgery.

**Table 3:** Overall survival rates for different patients in four studies.

OS	Bulky N2+/PAN-		Bulky N2-/PAN+		Bulky N2+/PAN+	
	3-y	5-y	3-y	5-y	3-y	5-y
**JCOG0001 (n=49)**	37.5%	29.2%	22.2%	22.2%	25%	18.8%
**JCOG0405 (n=47)**	82.7%	73.4%	64.3%	57.1%	20%	20%
**JCOG1002 (n=52)**	62.1%	57.1%	50%	35.7%	77.8%	77.8%
**This study (n=83)**	77.1%	71.6%	50.0%	50.0%	7.4%	0%

For patients in the Bulky N2+/PAN- group, the 3-year and 5-year OS rates in this study were 77.1% and 71.6%. The 3-year and 5-year OS rates in the JCOG0001 study were 37.5% and 29.2%. The 3-year and 5-year OS rates in the JCOG0405 study were 82.7% and 73.4%. The 3-year and 5-year OS rates in the JCOG1002 study were 62.1% and 57.1%. We found that the 5-year OS rate in this study (71.6%) were similar to those in the JCOG0405 (73.4%) and higher than those in the JCOG0001 (29.2%) and JCOG1002 (57.1%). On the other hand, there were obviously fewer surgical complications in this study than in the JCOG studies (**Table 4**, P<0.001). Besides, in a previous study of PCT followed by D2 lymphadenectomy for GC patients with PAN metastases, only one of the 28 patients had surgical complication (25). These results showed D2 gastrectomy alone is safer than surgery plus PAND. Based on the above analysis, we consider D2 lymphadenectomy alone is suitable for GC patients with only bulky N2 metastases after PCT. Of course, further clinical studies are still needed to investigate whether D2 lymphadenectomy alone or the combination of D2 lymphadenectomy and PAND has a better survival benefit for these patients.

**Table 4:** Surgical complications in all operated patients.

Characteristics	JCOG0001(n = 47)	JCOG0405(n = 49)	JCOG1002(n = 46)	This study(n = 83)	P
**Leakage**	1	3	2	2	
**Pancreatic fistula**	6	11	9	0	
**Abdominal abscess**	2	8	5	0	
**Pneumonia**	2	2	4	0	
**Ileus**	0	0	1	0	
**Wound infection**	2	0	2	0	
**Anastomotic stenosis**	1	0	1	0	
**Cardiac failure**	1	0	0	0	
**Renal dysfunction**	1	0	0	0	
**Thromboembolic event**	0	2	2	0	
**Atelectasis**	0	3	0	0	
**Other**	6	11	11	0	
**Total**	22	40	37	2	<0.001*

*χ^2^ test (compares the counts of categorical responses between 2 or more independent groups).

For patients with only PAN metastases, the 3-year and 5-year OS rates in this study were 50.0% and 50.0%, respectively. The 3-year and 5-year OS rates in the JCOG0001 study were 22.2% and 22.2%. The 3-year and 5-year OS rates in the JCOG0405 study were 64.3% and 57.1%. The 3-year and 5-year OS rates in the JCOG1002 study were 50% and 35.7%. We found that the 5-year OS rate in this study (50%) was similar to that in the JCOG0405 study (57.1%) and higher than those in the JCOG0001 (22.2%) and JCOG1002 (35.7%). This difference may be due to the number of false positives in patients with only PAN metastases. A previous study on PAND for GC with 1–3 involved PANs showed that the actual PAN metastases rate was 30.4% (33).

For patients with bulky N2 and PAN metastases, the 3-year and 5-year OS rates in this study were 7.4% and 0%. The 3-year and 5-year OS rates in the JCOG0001 study were 25% and 18.8%. The 3-year and 5-year OS rates in the JCOG0405 study were 20% and 20%. The 3-year and 5-year OS rates in the JCOG1002 study were 77.8% and 77.8%. We found that the 5-year OS rate in this study (0%)were lower than those in the JCOG0001 (18.8%), JCOG0405 (20%), and JCOG1002 (77.8%). According to the pathological evaluation, more patients in our study achieved grades of ypT0 and ypN0 and PCR than those in the JCOG0001, JCOG0405 and JCOG1002 studies (**Table 5**) which showed that the chemotherapy regimen in this study was effective and feasible. Therefore, the significant difference in survival for patients with bulky N2 and PAN metastases may be mainly due to the different surgeries. Therefore, we consider D2 lymphadenectomy alone is not suitable for GC patients with bulky N2 and PAN metastases after PCT.

**Table 5:** Pathological evaluation for four studies.

Characteristics	JCOG0001 (n = 47)	JCOG0405 (n = 49)	JCOG1002 (n = 46)	This study (n = 83)	P
**Depth of tumor invasion**					<0.001*
ypT0	1 (2.1%)	2 (4.1%)	1 (2.2%)	12 (14.5%)	
ypT1	3 (6.4%)	7 (14.3%)	8 (17.4%)	6 (7.2%)	
ypT2	18 (38.3%)	23 (46.9%)	9 (19.6%)	14 (16.9%)	
ypT3	19 (40.4%)	16 (32.7%)	21 (45.6%)	10 (12.0%)	
ypT4	6 (12.8%)	1 (2.0%)	7 (15.2%)	41 (49.4%)	
**Nodal status**					<0.001*
ypN0	1 (2.1%)	8 (16.3%)	10 (21.7%)	26 (31.3%)	
ypN1	7 (14.9%)	5 (10.2%)	8 (17.4%)	14 (16.9%)	
ypN2	9 (19.1%)	21 (42.9%)	9 (19.6%)	14 (16.9%)	
ypN3	30 (63.8%)	15 (30.6%)	19 (41.3%)	29 (34.9%)	
**Pathological response**					<0.001*
Grade 0	6 (12.8%)	3 (6.1%)	3 (6.5%)	4 (4.8%)	
Grade 1a	33 (70.2%)	19 (38.8%)	17 (37.0%)	12 (14.5%)	
Grade 1b	2 (4.3%)	13 (26.5%)	8 (17.4%)	10 (12.0%)	
Grade 2	5 (10.6%)	13 (26.5%)	17 (37.0%)	45 (54.2%)	
Grade 3	1 (2.1%)	1 (2.0%)	1 (2.1%)	12 (14.5%)	

*χ^2^ test (compares the counts of categorical responses between 2 or more independent groups).

There are some limitations to this study. This study was a retrospective study. Prospective studies are needed to further confirm the results. In addition, we did not have detailed data from the JCOG studies, and we can compare only our results with the data presented in their published articles. Since these studies were not conducted at the same time, there were some biases that could not be avoided. However, this study not only confirms our practice of not performing PAND in GC patients with only bulky N2 metastases, but also reminds us of the importance of PAND in the treatment of GC patients with bulky N2 and PAN metastases.

In conclusion, we consider D2 lymphadenectomy alone is suitable for GC patients with only bulky N2 metastases after PCT. However, for GC patients with bulky N2 and PAN metastases, D2 lymphadenectomy alone perhaps is not suitable. These patients need D2 lymphadenectomy plus PAND after PCT (**Figure 1B**).

## Data Availability Statement

The original contributions presented in the study are included in the article/supplementary material. Further inquiries can be directed to the corresponding authors.

## Ethics Statement

The studies involving human participants were reviewed and approved by the Human Ethics Committee of Shanghai Jiao Tong University School of Medicine Ruijin Hospital. The patients/participants provided their written informed consent to participate in this study.

## Author Contributions

WX conceived and designed the study, analyzed the data and wrote the paper. WL participated in the design of the study and edited the final paper. LW, CH, SL, ZN, ZH, ZLZ, ZY, BS, and YZ collected the clinicopathological factors of all patients. RF, CL, XY, and MC followed up the patient’s survival status. CY revised the paper. MY and ZGZ gave professional guidance. All authors contributed to the article and approved the submitted version.

## Funding

This work was supported by National Natural Science Foundation of China NO. 81772518 and clinical research project of Ruijin Hospital, Shanghai Jiao Tong University School of Medicine (DLY201602 and 2018CR003).

## Conflict of Interest

The authors declare that the research was conducted in the absence of any commercial or financial relationships that could be construed as a potential conflict of interest.

## Publisher’s Note

All claims expressed in this article are solely those of the authors and do not necessarily represent those of their affiliated organizations, or those of the publisher, the editors and the reviewers. Any product that may be evaluated in this article, or claim that may be made by its manufacturer, is not guaranteed or endorsed by the publisher.
